# Incidence and risk factors of stress urinary incontinence after laparoscopic hysterectomy

**DOI:** 10.1186/s12905-024-02942-2

**Published:** 2024-02-08

**Authors:** XiaoHong Qian, DongFang Ren, liJuan Gu, Cong Ye

**Affiliations:** 1 Department of Gynecology, Taicang Affiliated Hospital of Soochow University (The First People’s Hospital of Taicang), Taicang, Jiangsu Province 215400 China; 2 Department of Obstetrics, Taicang Affiliated Hospital of Soochow University (The First People’s Hospital of Taicang), Taicang, Jiangsu Province 215400 China

**Keywords:** Hysterectomy, Stress urinary incontinence, Risk factor analysis

## Abstract

**Objective:**

To observe the long-term effects of total hysterectomy on urinary function, evaluate the effects of preoperative nutritional status, urinary occult infection, and surgical factors on the induction of postoperative stress urinary incontinence (SUI), and explore the incidence and risk factors of SUI.

**Study design:**

From January 2017 to December 2017, 164 patients with benign non-prolapsing diseases who underwent a laparoscopic total hysterectomy in the First People's Hospital of Taicang were selected as the analysis objects. The International Incontinence Standard Questionnaire for Female Lower Urinary Tract Symptoms (ICIQ-FLUTS) and Pelvic Floor Impact Questionnaire-short version 20 (PFDI-20) were used for telephone follow-up to subjectively assess the urinary function of patients, collect their medical records, and statistically analyze the number of postoperative SUI cases. Logistic multivariate analysis was used to analyze the influencing factors of postoperative female SUI, presented as adjusted odds ratios with 95% confidence intervals.

**Results:**

Only 97 out of 164 patients completed the ICIQ-FLUTS and PFDI-20 questionnaires. Among these participants, 28 patients (28.86%) were diagnosed with SUI (study group), while 69 patients (71.13%) were classified as women without SUI (control group). The age, menopause, parity ≥ 2 times, Body mass index (BMI) ≥ 28 kg/m^2^, neonatal weight ≥ 4000 g, history of chronic cough, preoperative hemoglobin ≤ 100 g/L, preoperative urine bacteria ≥ 100u/L, preoperative uterine volume ≥ 90 cm^3^, intraoperative blood loss, and operation time of the study group were compared with those of the control group. The differences were statistically significant (*P* < 0.05). Further Logistic multivariate analysis showed that menopause, preoperative hemoglobin ≤ 100 g/L, preoperative urine bacteria ≥ 100u/L, uterine volume ≥ 90 cm^3^, history of chronic cough, BMI ≥ 28 kg/m^2^ were risk factors for postoperative SUI in patients undergoing hysterectomy (*P* < 0.05).

**Conclusions:**

Hysterectomy for benign non-prolapse diseases has a long-term potential impact on the urinary system of patients, and the risk of postoperative SUI increases. The main risk factors of SUI are parity, menopausal status, obesity, preoperative nutritional status, and occult infection of the urinary system.

## Introduction

Hysterectomy is one of the most common gynecological surgeries and the second most common operation after cesarean section in the world. According to incomplete statistics, millions of patients undergo hysterectomies every year worldwide [1]. Nearly 90% of hysterectomies are the radical treatment for benign diseases such as cervical lesions, endometrial lesions, uterine fibroids, and adenomyosis [2]. According to a study published in The Lancet, hysterectomy may affect lower urinary tract function, and its most common long-term sequelae are SUI [3]. The pathological mechanism of the specific pathogenesis is believed to be related to the injury of pelvic floor support structure I during the operation. In a total hysterectomy, the sacral and cardinal ligaments around the uterus, which mainly support and fix the uterus, were cut off, resulting in changes in the anatomical position of the bladder and the angle between the urethra. A nationwide, population-based cohort study conducted in Sweden over a period of 30 years showed that hysterectomy for benign indications significantly increased the risk of subsequent surgery for SUI, with a 6.3-fold increase in the risk of severe SUI [4].

Despite the high incidence of SUI after hysterectomy, only 9% of affected women seek treatment in time, and many more women miss a good opportunity for prevention and early intervention.This study aims to observe the long-term effects of hysterectomy on urinary function and explore the prevalence and risk factors of postoperative urinary incontinence (SUI), to achieve early detection and treatment of SUI after hysterectomy and improve the quality of life and physical and mental health of women.

## Material and methods

Our study was a retrospective case–control study conducted at a single center, involving data collection and analysis. The focus of this study was to investigate the relationship between preoperative factors and the occurrence of SUI after hysterectomy, rather than establishing a causal relationship. All methods were carried out in accordance with relevant guidelines and regulations or Declaration of Helsinki. This study was approved by the Ethics Committee of the First People's Hospital of Taicang and the informed consent forms were signed for all enrolled patients. Our study was registered with Trial registration: 2023-KY-505. The data were derived from 164 patients who underwent laparoscopic total hysterectomy due to non-prolapse benign diseases (confirmed by postoperative pathological results) in the First People's Hospital of Taichang from January 2017 to December 2017. Assuming a 20% incidence of SUI, we would need to recruit at least 80 patients to detect a 25% difference in risk (α = 0.05, β = 0.2). Considering factors such as loss to follow-up rate and data quality, we finally decided to recruit 164 patients to be included in the study.The youngest was 35 years old, the oldest was 63 years old, and the average age was (49.71 ± 12.98) years old. There were 87 cases of uterine fibroids, 53 cases of adenomyosis, 13 cases of adenomyoma, 7 cases of cervical high-grade intraepithelial lesions, and 1 case of abnormal uterine bleeding. Three cases had atypical endometrial hyperplasia.Among them,The reasons for hysterectomy in postmenopausal patients were as follows:

- (1) The continuous enlargement of uterine fibroids after menopause.
- (2) Cervical biopsy showed a high-grade cervical intraepithelial lesion, and the pathology after cervical conization showed a positive endocervical margin.
- (3) When cervical biopsy indicated high-grade cervical intraepithelial lesion, but cervical atrophy could not complete cervical conization, hysterectomy was performed when pelvic MRI was performed to fully evaluate and exclude malignant lesions.

Patients aged 30 to 65 years undergoing laparoscopic total hysterectomy for non-prolapsing benign diseases (e.g., uterine fibroids, adenomyosis, adenomyoma, abnormal uterine bleeding, cervical epithelial neoplasia, and atypical endometrial hyperplasia) were included. Standardized surgical procedures were used in this study to perform hysterectomies and to ensure that each surgeon followed the same procedure. The surgical procedure included general anesthesia, laparoscopy, and hysterectomy. All operations were performed by three doctors with many years of surgical experience, one of whom was the primary surgeon and the other two as assistants. Before the procedure, the surgeon explained the procedure and precautions in detail and provided training and demonstration to other doctors. Patients with previous urinary system diseases, history of pelvic surgery, complicated with pelvic organ prolapse or urinary incontinence, complicated with severe endometriosis, pelvic adhesions, and complicated with serious underlying diseases (such as heart disease and malignant tumors in other parts of the body) were excluded.

The validated Chinese version of the International Incontinence Standard Questionnaire for Female Lower Urinary Tract Symptoms (ICIQ-FLUTS) and Urogenital Distress Inventory 6 (UDI 6) were used to follow up with the eligible patients by telephone questionnaire to subjectively evaluate the patient's urinary function and complete the urinary function evaluation survey. Sixty-seven of 164 patients were lost to follow-up. Patients who could not be contacted were followed up by telephone at least 3 times at different times, and excluded when they could not be contacted.

The follow-up patients were divided into a study group (SUI group) and a control group (non-SUI group) based on the occurrence of SUI. The relevant clinical data of the patients were collected, and the number of SUI cases after surgery was statistically analyzed. Logistic multivariate analysis was employed to examine the risk factors of SUI in female patients after surgery. In our regression analysis, we included age as a covariate to account for its potential influence on the results.

The retrospective case–control study included ①general information about the patients: age, type of primary disease, family history of SUI, gravidity, parity, the weight of the delivered newborn, menstrual cycle, BMI index, chronic cough, and constipation history. ②Preoperative imaging features uterine volume (uterine volume (length * width * thickness *0.5236) cm3; ③Preoperative laboratory indicators: nutritional status (hemoglobin, albumin), infection status (white blood cell count); Urinary system status (urine white blood cells, urine bacteria) ④intraoperative blood loss, operation time; ⑤Postoperative retention time of catheterization, urine red blood cells and urine occult blood, a total of 20 clinical indicators that may affect postoperative SUI in patients with hysterectomy were included. Among them, Chronic cough refers to a persistent cough that lasts for an extended period, typically more than one years. A history of constipation refers to a prolonged condition characterized by infrequent bowel movements or difficulty in passing stools, typically more than one years. The data were collected by 3 students independently, and the quality control was completed by experts in epidemiology and statistics.

Specific cut-off values were set for some clinical indicators of SUI. The reason for choosing age 50 as the cutoff value is that the risk of SUI increases with age in women. Women over the age of 50 are more likely to have pelvic floor dysfunction than younger women. Setting parity as 2 as the cutoff value is because multiple pregnancies and childbirths can cause damage to the pelvic floor muscles and ligaments, increasing the risk of SUI. Women with a parity of 2 or more are more likely to experience pelvic floor dysfunction compared to those who have not given birth. The reason for setting BMI (Body Mass Index) as 28 kg/m^2^ as the cutoff value is that high BMI is associated with an increased risk of pelvic floor dysfunction and urinary incontinence. Excessive weight puts strain on the pelvic floor muscles and ligaments, leading to their weakened function and an increased risk of urinary incontinence. Setting a birth weight of 4000 g as the cutoff value is because delivering a macrosomic infant (weighing over 4000 g) can increase the stress on the pelvic floor muscles and ligaments, leading to their weakened function and an increased risk of urinary incontinence. Setting preoperative hemoglobin level as 100 g/L as the cutoff value is because low hemoglobin levels may be associated with weakened function of the pelvic floor muscles and ligaments. Hemoglobin below 100 g/L is more likely to lead to tissue hypoxia, affecting the normal contraction and support function of the pelvic floor muscles. Setting preoperative urine bacteria count as 100u/L as the cutoff value is because a high bacteria count in the urine may be related to urinary tract infections, which are a common factor in causing urinary incontinence. Setting preoperative uterine volume as 90cm^3^ as the cutoff value is because a larger uterine volume(Normal uterine volume is about 90cm^3^) can increase the stress on the pelvic floor muscles and ligaments, leading to their weakened function and an increased risk of urinary incontinence.

Pelvic floor and urinary function evaluation: The ICIQ-FLUTS and UDI-6 were used to assess the symptoms and severity of postoperative urinary incontinence and its impact on quality of life [5]. Diagnostic criteria of SUI: According to the definition criteria of the International Urinary Continence Society (ICS) in 2002, urinary incontinence was defined as an involuntary outflow of urine with or without any cause. This corresponds to question 10 of the ICIQ-FLUTS questionnaire or question 17 of the UDI-6 questionnaire. Question 10 of the ICIQ-FLUTS is "How often do you leak urine?" The possible responses are: ①Never ②Less than once a week ③Once or more per week, but not every day ④Once or more per day. SUI related questions on the UDI-6 questionnaire: Urine leakage related to physical activity? (walking, running, laughing, sneezing, coughing)The possible responses are: ①Not at All ②A Little Bit ③Moderately ④Greatly. If the answer to both was 0, it was defined as no SUI, and vice versa. For those patients with SUI indicated by the questionnaire, we continued to perform stress and Bonney tests to further confirm the diagnosis. The stress test involves examining the patient's bladder in the supine position when it is filled. The physician instructs the patient to cough while observing the urethral opening. If involuntary urine leakage occurs with each cough, it indicates SUI. The Bonney test refers to the examiner putting the middle and index finger into the patient's vagina, on either side of the urethra's anterior wall, the fingertip is located at the junction of the bladder and urethra, elevating the bladder neck, and then performing the induced stress test. If the SUI phenomenon disappears, it is positive and can be diagnosed as SUI. At the same time, we also conducted a one-hour urine pad test as a supplement to the diagnosis, and considered ≥ 2 g as positive, with SUI.

### Statistical analyses

To determine the incidence of SUI after hysterectomy and its risk factors, we performed a statistic assessment. In this process, we used statistical methods based on the logistic regression model to process and analyze the data. First, we collected the relevant clinical data before and during the operation and the results of the questionnaire after the operation, and then cleaned and organized them. The logistic regression model was used to develop the prediction model, and the maximum likelihood method was used to estimate the model parameters. According to the statistical data of the observed indicators, the Mean Value plus or minus Standard Deviation ($\overline{x }\pm s$), case (*n*), and percentage (%) were used to represent the measurement data and count data, respectively. The effective data were input into SPSS18.0 software for statistical analysis, and the t value and × 2 test were performed. *P* < 0. 05 was considered statistically significant. Stepwise Logistic regression analysis was used to determine the risk factors of SUI after a total hysterectomy, and the inclusion criteria were *P* < 0.05. The statistically significant risk factors were included in the multivariate regression analysis, and the Logistic regression equation was used to analyze and screen the main risk factors of postoperative SUI.

## Results

### Univariate analysis of risk factors for SUI after laparoscopic hysterectomy

Total of 164 patients with benign non-prolapsed diseases who underwent laparoscopic hysterectomy was included in this study. ICIQ-FLUTS and PFDI-20 questionnaires were completed in 97 patients, with an effective rate of 59.1%. The mean follow-up time was 55.0 ± 6.8 months. In the study group, 28 (28.86%)patients with SUI were included, while the control group consisted of 69 (71.13%)women without SUI. All patients diagnosed with SUI were confirmed through Bonney and stress tests. As shown in Table 1, comparison of risk factors between the two groups showed that: the age of the study group was ≥ 50 years old, SUI family history, menopause, parity ≥ 2, BMI ≥ 28 kg/m^2^, neonatal weight ≥ 4000 g, history of chronic cough, preoperative hemoglobin ≤ 100 g/L, preoperative urine bacteria ≥ 100u/L, preoperative uterine volume ≥ 90cm^3^ there were significant differences in intraoperative blood loss and operation time between the two groups (*P* < 0. 05). In addition, a one-hour pad test was conducted to assess the severity of SUI in the study group. The results of the pad test revealed that the mean urine leakage in the study group was 15.6 ± 2.3 g during the one-hour duration. Among the 28 patients with SUI, 17 (60.7%) had a urine leakage of more than 10 g, indicating a significant degree of incontinence.

**Table 1:** Univariate analysis of risk factors for SUI after laparoscopic hysterectomy

**Risk factors**		**SUI group **(*n* = 28)	**Control group **(*n* = 69)	**Test value**	***P value***
**Age (years)**	≥ 50	8	20	25.525	0.032
	< 50	20	49		
**Primary disease type**	myoma of uterus	13	25	0.071	0.703
	adenomyosis	8	20		
	endometrioma	2	7		
	abnormal uterine bleeding	2	7		
	Cervical epithelial neoplasia	2	6		
	atypical hyperplasia of endometrium	1	4		
**Family history of SUI**	yes	2	5	1.037	0.123
	no	26	64		
**Parity (times)**	≥ 2	17	56	22.406	0.013
**Newborn birth weight ≥ 4000 g**	yes	22	40	10.198	0.024
	no	6	29		
**Menstrual cycle**	regularity	15	38	0.084	0.073
	irregularity	13	31		
**BMI index**	≥ 28 kg/ m^2^	20	36	16.970	0.037
	< 28 kg/ m^2^	8	33		
**History of chronic cough**	yes	23	39	21.529	0.045
	no	5	30		
	no	10	48		
**Pausimenia**	yes	24	34	20.079	0.034
	no	4	35		
**Preoperative uterine volume(cm**^**3**^**)**	≥ 90	22	38	21.531	0.041
	< 90	6	31		
**Preoperative hemoglobin(g/L)**	≥ 100	19	37	23.673	0.018
	< 100	9	32		
**Preoperative albumin(g/L)**	≥ 35	11	40	18.686	0.997
	< 35	17	29		
Preoperative blood white blood cell count(*10^**9**^**/L)**	> 9.5	15	38	0.754	0.463
	≤ 9.5	13	31		
**Preoperative urinary white blood cell count(/uL)**	> 28	16	35	0.535	0.618
**Preoperative urinary bacterial count(/uL)**	> 7	25	42	21.422	< 0.05
	≤ 7	3	27		
**Intraoperative blood loss(ml)**	≥ 100	23	43	17.735	0.033
	< 100	5	26		
**Time of operation(h)**	≥ 2	18	39	24.311	0.040
	< 2	10	30		
**Postoperative catheter retention time**	≥ 72 h	16	36	0.438	0.156
	< 72 h	12	33		
**Postoperative urine erythrocyte count**	≥ 30U/L	17	34	0.552	0.638
	< 30U/L	11	35		
**Hematuria**	≥ 2 +	15	37	0.505	0.762
	< 2 +	13	32		

### Multivariate analysis of risk factors for SUI after laparoscopic hysterectomy

As shown in Table 2, the statistically significant variables in the univariate analysis were subjected to Logistic multivariate analysis. Age, preoperative hemoglobin ≤ 100 g/L, preoperative urine bacteria ≥ 100u/L, uterine volume ≥ 90cm3, history of chronic cough, and BMI ≥ 28 kg/ m^2^ are risk factors for postoperative urinary incontinence in patients undergoing hysterectomy (*P* < 0.05).

**Table 2:** Multivariate analysis of risk factors for SUI after laparoscopic hysterectomy

Risk factors	Standard deviation	β	OR	95%CI	Wald*x*^*2*^value	*P* value
Age ≥ 50 years old	0.561	1.956	6.710	2.371–21.526	13.771	0.015
Family history of SUI	0.059	2.150	3.121	1.099–2.308	0.766	0.059
Parity ≥ 2 (times)	0.589	2.366	12.654	4.987–42.360	22.092	0.054
BMI index ≥ 28 kg/ m^2^	0.523	1.620	2.978	1.049–7.556	6.140	0.011
Preoperative hemoglobin ≤ 100 g/L	0.515	1.110	2.741	1.065–7.342	5.226	0.027
Preoperative urinary bacteria ≥ 100u/L	0.073	1.964	2.793	1.102–3.482	15.563	0.035
Intraoperative blood loss (ml)	0.690	3.188	4.951	3.780–24.511	0.881	0.752
Uterine volume ≥ 90cm^3^	0.558	2.630	5.227	1.969–8.870	6.558	0.018
Time of operation	0.052	1.099	1.083	0.960–2.471	0.687	0.75
History of chronic cough	0.316	1.498	4.003	2.256–4.040	11.427	0.032
Newborn birth weight ≥ 4000 g	0.459	2.066	3.389	2.960–9.991	12.528	0.066

## Discussion

SUI is the involuntary leakage of urine during sneezing, coughing, or any physical activity [6]. Urinary incontinence has been considered one of the five major diseases affecting human health. On the one hand, its symptoms limit women's daily life and activities and seriously affect their quality of life. On the other hand, it can lead to depression, depression, and loneliness, causing a huge psychological burden on patients, and then causing a series of social and health problems, and place a significant financial burden on health systems [7]. The prevalence of SUI depends primarily on the study population [8]. According to statistics from European and American countries, the incidence of SUI in women aged 40 years and older is about 15.9%. According to Chinese data, the overall incidence of SUI in adult women is about 18.9%, but it increases with age, and the incidence of SUI is reported to be as high as 28.0% in 50–59 years old. According to the China Development Report 2020: Development Trends and Policies of Population Aging in China, it is expected that close to 100 million elderly women will be troubled by SUI by 2050.

However, the incidence of female SUI is different in the population, and there are many influencing factors. In addition to age and race, pregnancy and childbirth and pelvic organ surgery have a great impact on the occurrence of SUI [9]. Brown et al. 's study on the risk of urinary incontinence after hysterectomy showed that the long-term risk of urinary incontinence after hysterectomy was increased, and for women aged about 60 years, the risk was as high as 60% [10]. Kudish et al. followed up 53 569 postmenopausal women with a history of hysterectomy and 38 524 postmenopausal women without a hysterectomy and found that hysterectomy significantly increased the risk of SUI and urge urinary incontinence 3 years after surgery [11]. A significant correlation between hysterectomy and severity of SUI was also confirmed by the urodynamic measurement of Valsalva leak point pressure (VLPP). Compared with the general population, the mean VLPP of patients with a history of hysterectomy was significantly lower, and the risk of severe SUI increased by 6.3 times in patients with hysterectomy [12]. Our study found that the 5-year incidence of SUI after a hysterectomy was 28.86%, which was significantly higher than the overall incidence of SUI in adult women. It can be concluded that a hysterectomy may affect the function of the lower urinary tract system, leading to an increased incidence of SUI. The specific pathogenesis of this disease is believed to be related to the injury of pelvic floor support structure I during the operation. In a total hysterectomy, the sacral and cardinal ligaments around the uterus were severed, and the pubovesical and cervical ligaments were also cut, which led to changes in the anatomical position of the bladder, the angle between the urethra, and the mobility of the bladder neck [13, 14].

A prospective study on the incidence of urinary incontinence after a total hysterectomy to analyze the risk factors of urinary incontinence after a total hysterectomy found that the risk of urinary incontinence was the highest within three years after a total hysterectomy, and the risk factors were high BMI, old age, vaginal hysterectomy [15]. However, Al-Mehaisen et al. Conducted a 10-year follow-up of patients after a total hysterectomy and found that the occurrence of urinary incontinence after a hysterectomy was significantly related to age [16]. The above studies mainly focused on the general data of patients and ignored the effect of surgical trauma on the urinary system. Surgical trauma during a hysterectomy can increase the risk of SUI by disrupting urethral support or urethral sphincter innervation [17]. In this study, a case–control study was conducted on patients who underwent laparoscopic sub hysterectomy in the First People's Hospital of Taicang in 2017. Univariate and multivariate Logistic regression analysis showed that age, preoperative hemoglobin ≤ 100 g/L, preoperative urine bacteria ≥ 100u/L, uterine volume ≥ 90cm3, history of chronic cough, and BMI ≥ 28 kg/ m^2^ were the risk factors for postoperative SUI.In addition to the risk factors mentioned in the literature and guidelines, this study also included the risk factors that may affect the recovery of patients and induce SUI before, during, and after a hysterectomy. The effects of preoperative nutritional status, occult urinary tract infection, and surgical factors on the induction of SUI were evaluated. This study found that in addition to age, obesity, and other risk factors, the main risk factors of postoperative SUI include poor preoperative nutritional status, giant uterus, and occult infection of the urinary system.

Our findings revealed several important factors that contribute to the development and severity of SUI. Firstly, age was identified as a significant risk factor for SUI. This is consistent with previous studies that have shown an increased prevalence of SUI with advancing age. The weakening of pelvic floor muscles and decreased estrogen levels in postmenopausal women are believed to play a role in the age-related increase in SUI. Secondly, obesity emerged as another important risk factor for SUI. Excess body weight and increased abdominal pressure can put a strain on the pelvic floor muscles, leading to a higher risk of urinary leakage. Our study supports the existing evidence highlighting the association between obesity and SUI. Thirdly, our study revealed that chronic coughing, such as in patients with chronic obstructive pulmonary disease (COPD), is a risk factor for SUI. The repeated increase in intra-abdominal pressure during coughing episodes can strain the pelvic floor muscles and contribute to urinary leakage. Healthcare providers need to consider this factor when assessing and managing patients with SUI. Fourthly, elevated preoperative urine bacteria levels (≥ 100u/L) were also identified as a risk factor for postoperative UI. Urinary tract infections (UTIs) can cause inflammation and irritation of the bladder, leading to UI symptoms. Preoperative screening for and treatment of UTIs may be crucial in reducing the risk of postoperative SUI. Fifthly, Uterine volume ≥ 90cm3 was found to be associated with an increased risk of postoperative UI. A larger uterus may exert greater pressure on the bladder and pelvic floor muscles, leading to UI symptoms. Surgical techniques that minimize trauma to the pelvic floor and adequate postoperative care may help mitigate this risk in patients with larger uterine volumes.

While the exact mechanisms linking low hemoglobin levels to SUI following hysterectomy are not yet fully understood, several hypotheses can be proposed. Firstly, anemia may impair the healing process and tissue repair following surgery, including the pelvic floor muscles. Delayed or compromised tissue healing may lead to weakened pelvic floor support and subsequent urinary incontinence. Secondly, low hemoglobin levels can be indicative of underlying health conditions or comorbidities that may also contribute to SUI. For example, anemia may be associated with chronic diseases, such as renal dysfunction or cardiovascular disorders, which can independently affect pelvic floor function. It is important to consider these confounding factors when evaluating the relationship between low hemoglobin levels and SUI. It is worth noting that our findings regarding the association between preoperative hemoglobin levels ≤ 100 g/L and SUI following hysterectomy are based on our study. However, further research is needed to validate and elucidate this relationship, as the existing evidence is limited. In conclusion, our study suggests that preoperative hemoglobin levels ≤ 100 g/L may be a risk factor for SUI following hysterectomy. Further research is needed to better understand the underlying mechanisms and establish appropriate interventions to mitigate this risk. Clinicians should be aware of this potential association and consider preoperative anemia as part of the comprehensive assessment and management of patients undergoing hysterectomy.

The strength of this study is that the risk factors that may affect the recovery of patients and induce SUI before, during, and after hysterectomy were included, and the effects of preoperative nutritional status, occult urinary tract infection, and surgical factors on the induction of postoperative SUI were evaluated. However, we currently have a relatively small follow-up population and a high rate of loss to follow-up. In the next study, we will improve the patient follow-up information and further expand the data volume to conduct telephone follow-up and objective diagnosis and evaluation of all hysterectomy patients from 2017 to 2022. Hysterectomy can increase the risk of postoperative urinary incontinence. Preoperative nutritional status should be improved, the size of uterine lesions should be controlled, and long-term urinary function changes should be paid attention to after surgery.

## Data Availability

The datasets used and/or analysed during the current study available from the corresponding author on reasonable request.
